# The Different Effects of Atorvastatin and Pravastatin on Cell Death and PARP Activity in Pancreatic NIT-1 Cells

**DOI:** 10.1155/2016/1828071

**Published:** 2016-11-27

**Authors:** Ya-Hui Chen, Yi-Chun Chen, Chin-San Liu, Ming-Chia Hsieh

**Affiliations:** ^1^Diabetes Research Laboratory, Vascular and Genomic Center, Changhua Christian Hospital, Changhua, Taiwan; ^2^Institute of Biochemistry and Biotechnology, Chung Shan Medical University, Taichung, Taiwan; ^3^Vascular and Genomic Center, Changhua Christian Hospital, Changhua, Taiwan; ^4^Department of Neurology, Changhua Christian Hospital, Changhua, Taiwan; ^5^Graduate Institute of Integrative Medicine, China Medical University, Taichung, Taiwan; ^6^Division of Endocrinology and Metabolism, Department of Internal Medicine, Changhua Christian Hospital, Changhua, Taiwan

## Abstract

Statins have been widely used drugs for lowering low-density lipoprotein and for preventing heart attack and stroke. However, the increased risk for developing diabetes during extended stain use and the molecular mechanisms remain unclear. The objective of this study was to elucidate the signaling pathway and biological function between necrosis and autophagy induced by atorvastatin (AS) and pravastatin (PS). Here we observed that atorvastatin (AS) can increase intracellular reactive oxygen species (ROS) and induce necrotic cell death and autophagy in NIT-1 cells, whereas pravastatin (PS) does not cause ROS and cell death but also induces autophagy. PARP1 exhibited a dual role in modulating necrosis and autophagy in AS- and PS-treated NIT-1 cells through RIP1-RIP3-MLKL pathway and PARP1-AMPK-mTOR pathway. Lastly, AS treatment induced mitochondrial morphology injury significantly more than PS treatment did. Thus, the PARP1 activation should be considered in the development of effective statin therapies for diabetes. Future studies may examine specific mechanisms and pathways in mitochondria, autophagy, and oxidative stress* in vivo*.

## 1. Introduction

Statins are among the most commonly prescribed drugs in medicine. Studies have shown that statins can inhibit the action of HMG-CoA reductase in the liver, thereby slowing down the cholesterol production process [1]. Clinical studies have also shown that statins can reduce the risk of heart attack, stroke, and death in patients with heart disease such as coronary artery disease and cardiovascular disease [2, 3]. In addition, researchers have reported that statins may reduce the risk of developing colon cancer, non-small lung cancer, pancreatic cancer, and esophageal cancer [4–7]. Although these drugs have a satisfactory safety record, the increased risk for developing new-onset diabetes mellitus during extended statin use has recently generated attention; this includes simvastatin (10%), atorvastatin (AS, 22%), and rosuvastatin (18%) [8–11]. The molecular mechanisms for these associations remain unclear.* In vivo* and* in vitro* studies have recently found that these statins reduced insulin sensitivity and pancreatic *β*-cell function, possibly because of the effect on Ca^2+^ channels in *β*-cells, glucose transporter 4 translocation, insulin receptor substrate-1 and insulin receptor expression or phosphorylation, adipocyte maturation or differentiation, isoprenoid and coenzyme Q10 biosynthesis, and adiponectin and leptin levels [12, 13]. However, studies on pravastatin showed significant improvements in insulin sensitivity and *β*-cell function [14, 15]. Therefore, demonstrating the proposed mechanisms of statins therapy during the development of new-onset diabetes may be valuable for designing a new generation of statins without the aforementioned side effects.

Poly(ADP-ribosyl) polymerase-1 (PARP1) is a group of nuclear enzymes that has been associated with three different modes of cell death induced by DNA damage, namely, apoptosis, necrosis, and parthanatos. In addition, its overactivation results in NAD^+^/ATP energy depletion and eventually causes necrotic cell death [16–19]. However, studies have identified a novel function of PARP1 in mediating autophagy; thus, PARP1 exhibits a dual role in modulating autophagy and necrosis under oxidative stress and DNA damage [20–22].

Autophagy is an intracellular bulk degradation system for the removal of long-lived proteins and damaged organelles such as lysosomes. Studies have indicated that autophagy may prevent neurodegeneration, aging, and tumorigenesis [23–27]. By contrast, several studies have suggested that autophagy may trigger and mediate type II programmed cell death, which has been referred to as autophagic cell death [28, 29]. Autophagy has also been demonstrated as a prosurvival mechanism against cell death, especially under stress conditions such as starvation, metabolic stress, oxidative stress, and DNA damage [30, 31]. Thus, this study examined the signaling pathway linking PARP1 activation to autophagy under pharmaceutical stress, as well as the functional role of autophagy in pharmaceutical stress-mediated cell death.

## 2. Materials and Methods

### 2.1. Cell Culture

Mouse pancreatic NIT-1 cells were obtained from BCRC (Bioresource Collection and Research Center, Taiwan) and cultured in F-12K medium (Sigma-Aldrich, USA) supplemented with 10% FBS (Gibco, USA) at 37°C in a humidified 5% CO_2_.

### 2.2. Cell Viability Assay

Cells were seeded into a 96-well plate (6 × 10^4^/well) on F-12K medium and treated with statin (10–20 *μ*M, Merck Millipore, Germany) for 48 h, beginning 24 h after seeding. WST-1 solution (10 *μ*L, Roche, Germany) was added to each well and the cells were incubated for 1 h. The absorbance at 440 nm was measured using a microplate reader (FLUOstar Galaxy, Germany). In some experiments, the cells were pretreated with various inhibitors such as necrostatin-1 (Nec-1, an inhibitor of RIP1, 10 *μ*M, Enzo Life Sciences, USA), necrosulfonamide (NSA, an inhibitor of MLKL, 1.0 *μ*M, Millipore), bafilomycin A1 (Baf-A1, 2 nM, Sigma-Aldrich), 3,4-dihydro-5[4-(1-piperidinyl)butoxyl]-1(2H)-isoquinoline (DPQ, 60 *μ*M, Calbiochem), Z-Val-Ala-Asp-fluoromethylketone (zVAD-fmk, 2 *μ*M, BioVision, USA), and mTOR inhibitors (rapamycin, 20 nM, Adooq BioScience, USA) before statin treatment.

### 2.3. Measurement of Insulin and Interleukin-6 Assay

One-day cultured NIT-1 cells were treated with statin for 48 h. The supernatants were collected and the insulin concentrations were measured using Rat/Mouse Insulin 96-Well Plate Assay (Millipore) according to the manufacturer's instruction; the absorbance at 450 nm and 590 nm was measured by an ELISA reader (FLUOstar Galaxy). The limit of sensitivity of this assay is 0.1 ng/mL insulin. IL-6 was determined using the Mouse IL-6 ELISA kit (BD Biosciences, USA) according to the manufacturer's instruction; the absorbance at 450 nm was measured by an ELISA reader (FLUOstar Galaxy). The lower limit of detection for this ELISA was 3.8 pg/mL IL-6.

### 2.4. Lactate Dehydrogenase Assay

NIT-1 cells were seeded into a 96-well plate (6 × 10^4^/well) and cultured in statin-containing medium supplemented with 1% FBS for 48 h. After applying 100 *μ*L of the lactate dehydrogenase reaction mixture using the Cytotoxicity Detection Kit^Plus^ (Roche), the absorbance at 490 nm was measured by an ELISA reader (FLUOstar Galaxy): Cytotoxicity  (%) = (exp.  value − low  control)/(high  control − low  control)*∗*100.

### 2.5. Cell Cycle Analysis

NIT-1 cells were seeded in 12-well plates at 4 × 10^5^ cells/well and treated with statin for 48 h. Subsequently, the cells were collected, fixed with cold 70% absolute ethanol, and stored at −20°C overnight. Before detection, the cells underwent thawing and centrifugation and were washed with cold PBS. Subsequently, 1 mL of staining solution was added into each tube (20 *μ*g/mL of PI), followed by incubation in darkness for 30 min. Finally, the stained cells were analyzed using a flow cytometer (Cytomics FC 500, Beckman Coulter).

### 2.6. Annexin V and PI Staining

Cells were plated in a 6 cm culture dish at a seeding density of 5 × 10^6^ cells/dish. Following statin treatment, the medium was removed and washed twice with PBS. The cells were trypsinized, collected by centrifugation, resuspended in 500 *μ*L of 1X Annexin V Binding Buffer mixed with 1 *μ*L of Annexin V (BioVision) and 1 *μ*L of Propidium Iodide (BioVision), and incubated at room temperature for 15 min in darkness. The reaction volume was adjusted with the binding buffer to 1 mL. The cells were analyzed using a flow cytometer (Cytomics FC 500) with a single laser emitting excitation light at 488 nm.

### 2.7. ROS Measurement

The intracellular reactive oxygen species were assessed using the CellROX oxidative stress reagent (Life Technologies, USA) according to the manufacturer's instructions. Cells were plated in 12-well plates with a cell density of 8 × 10^5^ cells/well and treated with statin for 48 h. After statin treatment, the cells were stained with 5 *μ*M of CellROX green reagent and incubated in darkness for 30 min. After incubation, the stained cells were analyzed using the flow cytometer (Cytomics FC 500).

### 2.8. Confocal Laser Scanning Microscopy and Quantification

The 2.5 × 10^5^ cells were seeded in a 3.5 cm confocal dish and pretreated with various inhibitors before statin treatment. Following a 48 h treatment with statin, the cells were washed with PBS to remove dead cells and serum proteins. Immediately, treated or untreated NIT-1 cells were stained with an antibody against rabbit monoclonal AIF (1 : 400, Cell Signaling) or CYTO-ID Autophagy Detection Kit (Enzo Life Sciences, Farmingdale, New York, USA) and imaged via confocal microscopy using an Olympus FV1200 microscope (Tokyo, Japan). The images were converted to 8-bit grayscale and analyzed by the ImageJ software. The integrated density (IntDen) provided a measure of intensity proportional to total volume and was calculated using area. Values from six random images for each group were averaged for comparison.

### 2.9. Fluorescence Microscopy

The mitochondrial volume was determined using MitoTracker Red staining (Invitrogen, USA). The cells were cultured in a 6-well plate and pretreated with various inhibitors. After statin treatment, the cells were stained with MitoTracker Red and incubated at 37°C for 30 min in darkness. The cells were washed three times with PBS to remove unbound dye and recultured in F-12K medium. The fluorescence of the bound dyes was analyzed using an Olympus IX81 fluorescence microscope (Tokyo, Japan).

### 2.10. ATP Concentration Measurement

Cellular ATP synthesis was determined using the PhosphoWorks™ Luminometric ATP Assay Kit (AAT Bioquest, USA) according to the manufacturer's instructions. In brief, cells were seeded into 96-well plates and pretreated with inhibitors. After statin treatment, the cells were added with 100 *μ*L of ATP assay solution and incubated for 20 min at room temperature. The luminescence intensity was detected by an illuminometer (FLUOstar Galaxy).

### 2.11. Subcellular Fractionation

Nuclear and mitochondrial fractions were prepared from renal tissue using Nuclear Protein Isolation-Translocation Assay Kit (FIVEphoton Biochemicals, San Diego, CA, USA) and AllPure Mammalian Mitochondria Isolation Kit (AllBio Science Inc., Taiwan) according to the manufacturer's instructions.

### 2.12. Immunoprecipitation and Immunoblot Analysis

Whole-cell lysates were prepared using RIPA lysis buffer (Millipore, 20–188) and protein concentration was detected using a BCA protein assay kit (Thermo Scientific). A total of 150 *μ*g of cell lysates was mixed with anti-RIP3 (2 *μ*g, GeneTex, USA) or MagSi-protein A/G beads (50 *μ*L, MagnaMedics, Netherlands) at 4°C overnight. The beads were then collected using a magnet for 2 min, washed with PBST washing buffer three times, and subjected to elution with 40 *μ*L of 1x SDS loading buffer; the samples were incubated at 95°C for 10 min. Protein was separated using SDS-PAGE and transferred to 0.2 *μ*M PVDF membranes (Bio-Rad, USA). Blots were then probed with anti-RIP1, anti-RIP 3, anti-MLKL (GeneTex), monoclonal anti-AIF (Cell Signaling), polyclonal anti-COX4 (GeneTex), polyclonal anti-PCNA (GeneTex), monoclonal anti-PARP1, monoclonal anti-PAR (Cell Signaling), anti-phospho-AMPK*α* (Thr172), anti-AMPK*α*, anti-phospho-p70 S6 kinase (Thr389), anti-p70 S6 kinase (Cell Signaling, USA), MitoProfile Total OXPHOS rodent antibody cocktail (MitoSciences, OR), and mouse anti-GAPDH (Abcam, USA). Signals were obtained using an enhanced chemiluminescence kit (Millipore) and densitometry was performed using Fusion-Capt software (Vilber Lourmat, Fusion FX7, France).

### 2.13. Statistical Analysis

Statistical analyses were performed using SigmaPlot* t*-tests. Data are presented as mean ± SD from three independent experiments. A *P* value <0.05 was considered statistically significant.

## 3. Results

### 3.1. Atorvastatin Not Only Induces Pancreatic NIT-1 Cell Death But Also Reduces Insulin Secretion

**Figure 1:**
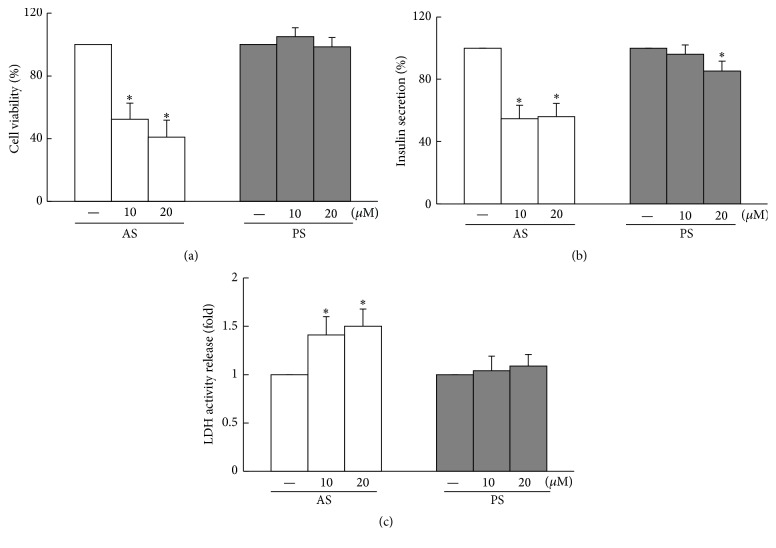
AS not only induces NIT-1 *β* cell death but also reduces insulin secretion. (a) Significantly dose-dependent cell death induced by AS in NIT-1 cells; no cell death in PS-treated cells. (b) Insulin secretion decreased considerably in NIT-1 cells after AS treatment and PS treatment with an increased dose. (c) The 48 h incubation of AS evidently increased the content of LDH in NIT-1 cells. Data are presented as mean ± SD from three independent experiments (^*∗*^ *P* < 0.05 compared to the untreated group,* t*-test). The value for the CON group was set at 1.

To determine the effects of statin on the cell viability of pancreatic *β* cells, NIT-1 cells were treated with various concentrations of AS or PS for 48 h by using the WST-1 assay. After treatment with AS (10 *μ*M and 20 *μ*M), the cell viabilities were determined to be approximately 52.3% and 41.0%, respectively, compared with the vehicle control. Moreover, after treatment with PS (10 *μ*M and 20 *μ*M), the cell viabilities were determined to be approximately 104.9% and 98.6%, respectively, compared with the vehicle control. Accordingly, treatment with AS results in the dose-dependent inhibition of cell viability in NIT-1 cells, but not treatment with PS (Figure 1(a)). Similarly, the reduction of cell viabilities under the 10 *μ*M and 20 *μ*M AS treatment could be associated with the decreased NIT-1 cell insulin secretion and increased lactate dehydrogenase (LDH) activity release, which is a cytosolic marker. Although the 20 *μ*M PS treatment resulted in less insulin secretion in the NIT-1 cells, it was insufficient to change the amount of LDH activity release (Figures 1(b) and 1(c)).

### 3.2. ROS-Induced Necrotic Cell Death Caused by AS Treatment

**Figure 2:**
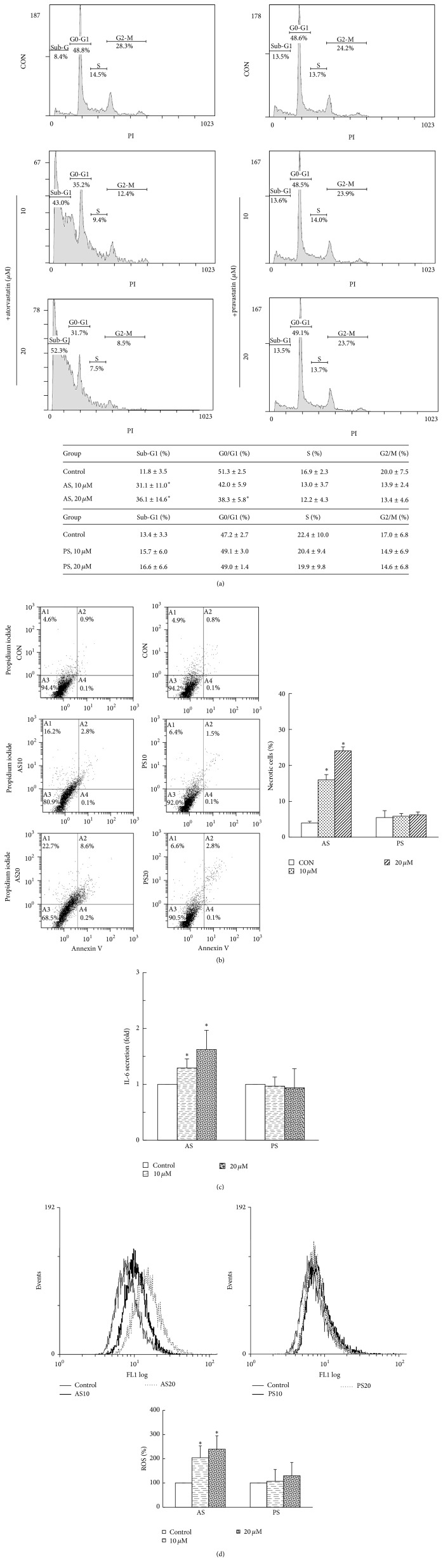
Treatment of AS in ROS-induced necrotic cell death. (a) The sub-G1 cell fractions were significantly dose-dependent and increased after exposure to AS; PS did not change cell cycle parameters. (b) Lower-left quadrants: viable cells. Lower-right quadrants: early apoptosis. Upper-left quadrants: necrotic cells. Upper-right quadrants: nonviable late apoptotic cells. An increased necrotic proportion of NIT-1 cells (Annexin V^−^PI^+^ cells) was observed after treatment with AS; no effect was observed in PS-treated cells. (c) IL-6 is a highly reliable marker of necrosis. IL-6 secretions in NIT-1 cells treated with AS; no IL-6 secretion was observed in PS-treated cells. The value for the CON group was set at 1. (d) Cells treated with AS or PS for 48 h were incubated with CellROX and measured using flow cytometry. The AS treatment of NIT-1 cells induced a dose-dependent increase of ROS production (^*∗*^ *P* < 0.05, compared to the untreated group, mean ± SD from three replicates).

To further examine the inhibitory effects of AS or PS on cell viability, cell death progression was examined using flow cytometric analysis. The treatment of NIT-1 cells with AS resulted in the significantly increased accumulation of cells in the sub-G1 phase (necrotic/apoptotic cells) in a dose-dependent manner, and the treatment with 20 *μ*M AS resulted in significantly fewer cells in the G0/G1 phase (*P* < 0.05). The PS treatment did not change cell cycle parameters (Figure 2(a)). Significantly increased necrosis phase (PI^+^ Annexin V^−^) percentages were found in proportion to the AS treatments (15.95% and 24.08% in the presence of 10 and 20 *μ*M AS, resp., *P* < 0.05), and the apoptotic phase (PI^−^ Annexin V^+^, data not shown) percentages were not significantly different among the groups. In addition, no significant difference was identified in the necrosis phase and apoptotic phase among the groups during the experimental period with PS treatment, compared to the vehicle control (Figure 2(b)).

To further confirm whether the NIT-1 cell death was caused by necrosis after AS treatment, the IL-6 expression level was measured using ELISA kits. The IL-6 expression level was significantly increased in the AS-treated groups 1.3- and 1.6-fold. However, no significant differences were found in the PS-treated groups compared with the vehicle control (Figure 2(c)). In addition, the AS treatment of NIT-1 cells induced a dose-dependent increase of intracellular ROS production (*P* < 0.05), whereas PS treatment did not result in significant differences among the groups (Figure 2(d)). The results indicate that AS treatment can diminish NIT-1 cell viability primarily by triggering necrosis and possibly increase production of LDH, IL-6, and ROS.

### 3.3. AS- and PS-Mediated Cell Deaths Are through Necrosis, Not Apoptosis and Parthanatos

**Figure 3:**
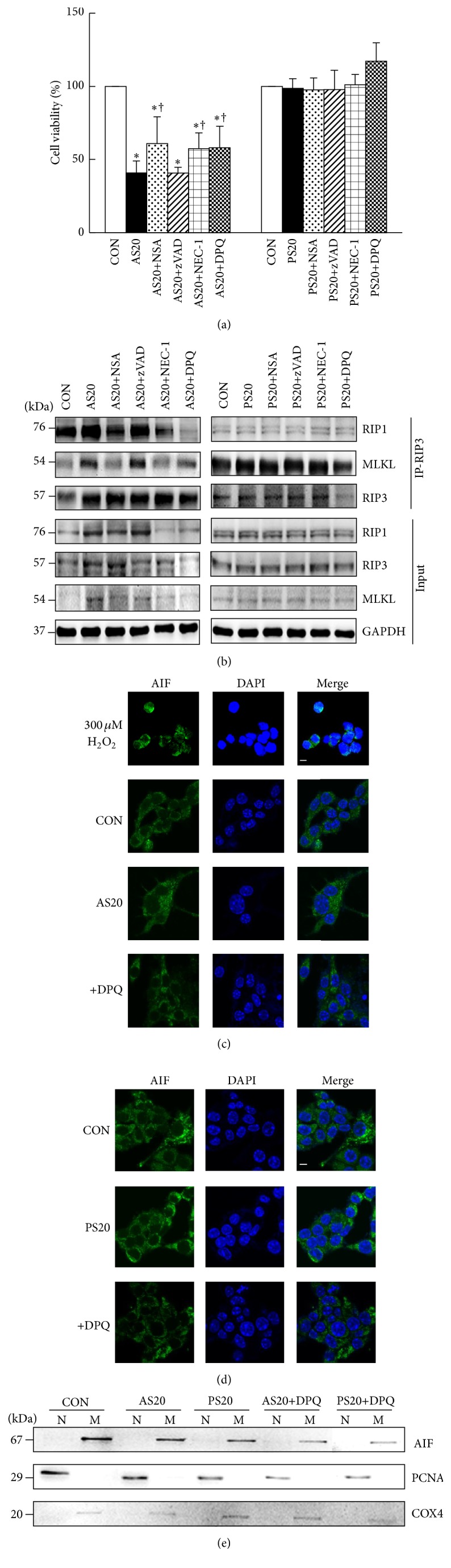
AS- and PS-mediated cell deaths are through necrosis, not apoptosis and parthanatos. (a) NIT-1 cells in AS (20 *μ*M) for 48 h exhibited decreased cell numbers. When pretreated with NSA or NEC-1, or DPQ, the cells exhibited an increased cell viability; the inhibitor zVAD did not change the percentage of NIT-1 cells. When pretreated with DPQ, the cells exhibited an increased cell viability in 20 *μ*M PS treatment but remained unchanged after NSA, NEC-1, and zVAD pretreatment. (b) The RIP1-RIP3-MLKL interaction was enhanced following necrosis induction. Whole-cell extracts were used for anti-RIP3 immunoprecipitation. The immune complexes were analyzed using Western blot analysis with the indicated antibodies. Whole-cell lysates (30 *μ*g, input) were analyzed by Western blotting for RIP3, RIP1, or MLKL. GAPDH is shown as the loading control. (c, d) Representative confocal images of AIF immunoreactivity (green) and nuclear DAPI staining (dark blue). AS and PS treatment did not induce AIF translocation to nucleus. Magnification: ×60. Scale bar: 20 *μ*M. 300 *μ*M H_2_O_2_ is included as positive control. (e) AIF is still normally localized to the mitochondrial (M) fraction in response to AS and PS treatment, not into nuclear (N) fraction. Proliferating cell nuclear antigen (PCNA) and cytochrome c oxidase subunit IV isoform 1 (COX4) were used as nuclear and mitochondrial marker proteins, respectively. ^*∗*^ *P* < 0.05, compared to the untreated group; ^†^ *P* < 0.05, compared to the only AS- or PS-treated group; mean ± SD from three replicates.

Next, we examined the inhibitor cell death response in the NIT-1 cells treated with AS and PS. NEC-1, NSA, and zVAD-fmk are typically used as necrosis and pan-caspase inhibitors* in vitro*, respectively [30, 31]. After 48 h, NSA and NEC-1 treatment increased the numbers of viable NIT-1 cells compared with the 20 *μ*M AS-treated group (61% versus 58% versus 41%, *P* < 0.05); zVAD-fmk treatment exhibited no effect on NIT-1 cell viability after the 20 *μ*M AS treatment. In addition, NSA, NEC-1, and zVAD-fmk treatments showed no significant differences when compared to the 20 *μ*M PS-treated group (98% versus 101% versus 98%, Figure 3(a)). By contrast, the necrosis induced by the AS treatment specifically caused the formation of the RIP1-RIP3-MLKL complex. The interactions between MLKL and RIP1 and RIP3 were significantly eliminated by NSA and NEC-1; thus, the RIP1 and MLKL signals appeared significantly reduced, and the RIP3 signal was significantly augmented in the presence of NSA and NEC-1. The PS treatment did not induce the formation of the RIP1-RIP3-MLKL complex (Figure 3(b)). Here, we investigated the upstream signaling pathways controlling the AS- and PS-mediated cell death. PARP1 activation has been suggested to be associated with the necrotic and parthanatos cell death progress [17, 19, 32, 33]. Thus, we used a specific chemical PARP inhibitor, DPQ. DPQ markedly reduced the statin-associated necrotic cell death in NIT-1 cells and significantly increased NIT-1 cell viability in the 20 *μ*M AS-treated group (58% versus 41%, *P* < 0.05, Figure 3(a)). Confocal microscopy and subcellular fractionation revealed that AS-treated or PS-treated cells did not induce apoptosis-inducing factor (AIF) translocation from mitochondria into the nucleus (Figures 3(c)–3(e), Figure S1A in Supplementary Material available online at http://dx.doi.org/10.1155/2016/1828071), suggesting that PARP1 activation plays crucial roles in AS-induced necrotic cell death, not via parthanatos.

### 3.4. Autophagy Plays a Prosurvival Role in AS- and PS-Mediated Cell Fate

**Figure 4:**
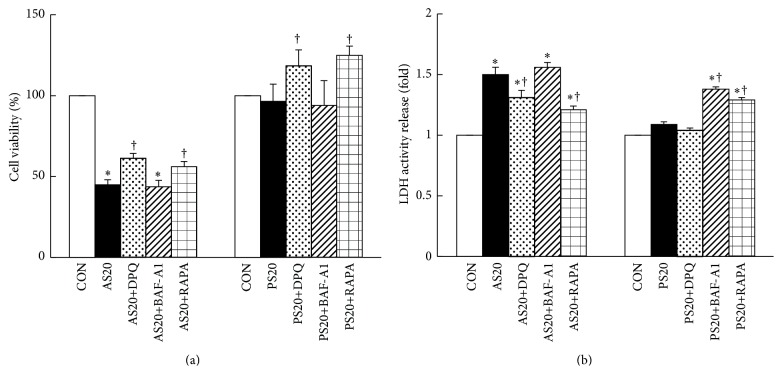
Autophagy plays a prosurvival role in AS- and PS-mediated cell outcome. (a) NIT-1 cells in AS exhibited substantially decreased cell viability. When pretreated with DPQ or RAPA, the cells increased in number; the autophagy inhibitor BAF-A1 mildly reduced cell viability. DPQ or RAPA exhibited increased cell viability in 20 *μ*M PS-treated cells, and BAF-A1 exhibited a mild decrease in cell viability. (b) LDH activity release in the NIT-1 cells after treatment with AS with or without DPQ, BAF-A1, and RAPA; DPQ and RAPA pretreatment significantly reduced LDH activity release; BAF-A1 significantly increased LDH activity release. Similarly, BAF-A1 significantly increased LDH activity release in NIT-1 cells treated with PS for 48 h, and RAPA pretreatment can attenuate BAF-A1-associated increase in LDH activity release. DPQ did not change the percentage of LDH activity release. The value for the CON group was set at 1. ^†^ *P* < 0.05, compared to the only AS- or PS-treated group; mean ± SD from three replicates.

Several studies have demonstrated that PARP1 exhibits a dual role in modulating autophagy and necrosis under oxidative stress and DNA damage, whereas PARP1 activation is associated with autophagy induction in AS and PS treatment. Rapamycin (RAPA) is an mTOR inhibitor and has been reported to promote autophagy processing, whereas bafilomycin A1 (BAF-A1) is an autophagy inhibitor [34]. The NIT-1 cell viability was significantly increased when the cells were treated with 20 *μ*M AS with RAPA, and the cell viability decreased mildly with BAF-A1 pretreatment, compared with the 20 *μ*M AS-treated group (56% versus 43% versus 45%, *P* < 0.05). Similarly, the NIT-1 cells with RAPA pretreatment showed considerably increased cell viability compared with the 20 *μ*M PS-treated group (125% versus 97%, *P* < 0.05, Figure 4(a), Figure S1B).

LDH activity release also was measured to determine the extent of NIT-1 cell death after exposure to inhibitor agents such as DPQ, BAF-A1, and RAPA. The treatment with DPQ and RAPA markedly reduced LDH release after the 20 *μ*M AS treatment (1.3- versus 1.2- versus 1.5-fold, *P* < 0.05), whereas the BAF-A1 treatment mildly increased LDH compared to the 20 *μ*M AS-treated group (Figure 4(b), Figure S1C). Similarly, the BAF-A1 treatment enhanced the LDH release content considerably, and the DPQ treatment only slightly reduced LDH activity release. RAPA treatment can attenuate BAF-A1-associated increase in the LDH activity release of NIT-1 cells after 20 *μ*M PS treatment (1.3- versus 1.4- versus 1.1-fold, *P* < 0.05). These results showed that autophagy acts as a cell survival mechanism in AS-induced necrotic cell death and that PS may be able to immediately induce autophagy to prevent NIT-1 cell death under pharmaceutical stress.

### 3.5. Role of PARP1 Expression in the Induction of AS and PS Autophagy

**Figure 5:**
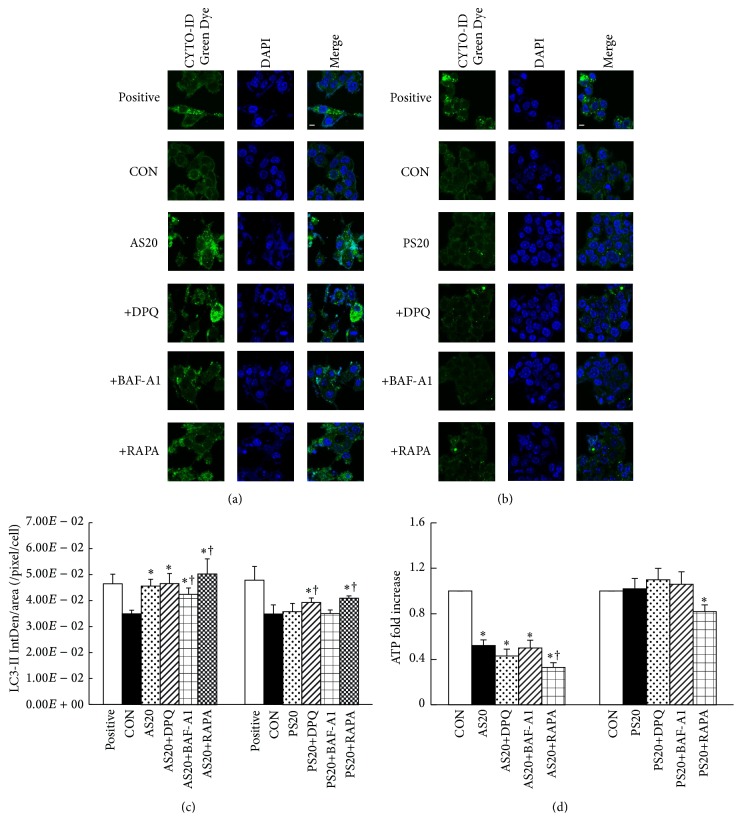
Role of PARP1 expression in the induction of AS and PS autophagy. (a) CYTO-ID® Green Dye staining revealed significantly increased LC3-II accumulation (green) in AS-treated NIT-1 cells compared with untreated cells; decreased LC3-II accumulation was observed after treatment with the BAF-A1 inhibitor; increased LC3-II accumulation was observed after treatment with the DPQ and RAPA inhibitors. Rapamycin is included as positive control. (b) No significant difference was found in the accumulation of LC3-II between control and PS-treated or PS+BAF-A1-treated NIT-1 cells, remaining high after DPQ or RAPA pretreatment. Chloroquine is included as positive control. Magnification: ×60. Scale bar: 20 *μ*M. (c) Quantification of the CYTO-ID Green Dye stain in NIT-1 cells per 600x fields for six fields per group. A significantly high number of LC3-II-positive cells were observed in the AS20, AS20+DPQ, and AS20+RAPA groups. (d) Cells treated with AS also showed a decreased ATP level, indicating that AS promoted ATP depletion in NIT-1 cells. DPQ significantly reduced the cellular ATP level. PS treatment for 48 h did not change the cellular ATP level and only mildly increased the ATP level after DPQ pretreatment. No significant difference was found compared with the untreated control group (^*∗*^ *P* < 0.05, compared to the untreated group; ^†^ *P* < 0.05, compared to the only AS- or PS-treated group; mean ± SD from three replicates). The value for the CON group was set at 1.

To evaluate whether PARP1 expression plays a crucial role in autophagic induction, NIT-1 cells were incubated in medium containing vehicle (control), statin, statin+DPQ (PARP1 inhibitor), statin+BAF-A1 (negative autophagy), and statin+RAPA (positive autophagy) for 48 h. The cells were then stained with CYTO-ID Green Dye Autophagy Detection Kit. Both AS-treated groups showed significantly stimulated further microtubule-associated protein 1A/1B-light chain 3- (LC3-) II accumulation, as autophagosome formation in NIT-1 cells compared with the vehicle control. AS+BAF-A1-treated group showed significantly reduced LC3-II expression compared with AS-treated group. No significant difference was found in the number of LC3-II between control and PS-treated or PS+BAF-A1-treated NIT-1 cells. Both DPQ- and RAPA-treated NIT-1 cells showed significantly bright green fluorescence compared with only AS-treated cells but only showed light green fluorescence when compared with PS-treated cells, respectively (Figures 5(a)–5(c), Figure S2A). These findings indicated that AS-treated cells significantly induced the autophagic activity; both DPQ- and RAPA-treated cells further enhanced the AS-treated increase of autophagy flux in NIT-1 cells. The PS-treated dose was to induce self-protective autophagy.

PARP1 is known as an energetically expensive process that leads to cellular ATP depletion and contributes to necrotic cell death [17]. Thus, ATP depletion was determined in AS-treated and PS-treated NIT-1 cells, and the results showed that the cellular ATP levels of AS-treated, AS+BAF-A1-treated, AS+DPQ-treated, and AS+RAPA-treated NIT-1 cells were significantly reduced compared with the untreated control (*P* < 0.05). However, DPQ- and RAPA-treated cells showed significantly increased AS-stimulated cellular ATP consumption, decreasing 0.86- and 0.66-fold (*P* < 0.05, Figure 5(d), Figure S2B). In addition, the RAPA-treated cells similarly exhibited significantly increased PS-stimulated cellular ATP consumption, but no significant difference was found in the cellular ATP levels among the PS-treated groups. The results clearly indicated that AS-induced PARP1 activation, mTOR suppression, and autophagy induction are mediated by inhibited PARP1 activation and enhance cellular ATP depletion.

### 3.6. Suppression of PARP1 Inhibits AS-Mediated Necrosis and AS-Induced Cell Death

**Figure 6:**
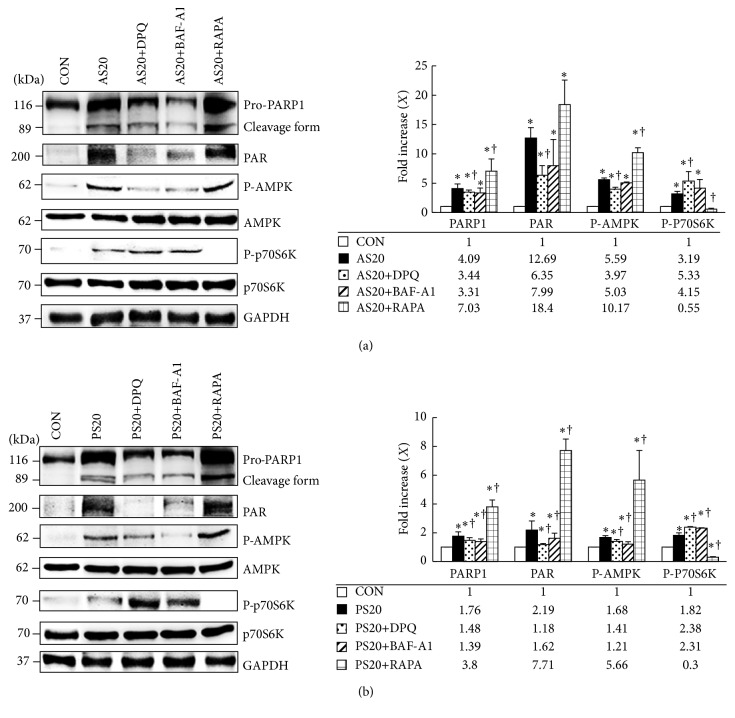
Suppression of PARP1 inhibits AS-mediated necrosis and AS-induced cell death. (a) NIT-1 cells cultured with AS treatment exhibited dependent activation of PARP1, PAR, and AMPK and reduced phosphorylated p70S6K compared with BAF-A1 negative autophagy control; the expression was reduced considerably compared to RAPA positive autophagy control. DPQ (PARP1 inhibitor) blocked AS-mediated PARP1, PAR, and AMPK upregulation. BAF-A1 markedly induced P-p70S6K formation; RAPA almost completely eliminated P-p70S6K. (b) PS treatment caused the significant upregulation of PARP1, PAR, and AMPK phosphorylation but a downregulation of p70S6K phosphorylation. DPQ significantly blocked PS-mediated PARP1, PAR, and AMPK upregulation. BAF-A1 also did markedly change P-p70S6K formation; RAPA almost completely eliminated P-p70S6K (^*∗*^ *P* < 0.05, compared to the untreated group; ^†^ *P* < 0.05, compared to the only AS- or PS-treated group; mean ± SD from three replicates).

After demonstrating the prosurvival role of autophagy and the role of PARP1 activation in AS-induced necrotic cell death, we investigated whether the signaling pathway of PARP1-AMPK-mTOR autophagy controls AS- and PS-mediated cell survival. Treatment with AS+DPQ markedly blocked AS-enhanced PARP1 cleavage and PAR expression, with a simultaneous decrease in the P-AMPK level and increase in P-p70S6K level (*P* < 0.05, Figure 6(a), Figure S3A). AS+BAF-A1 was expected to inhibit PARP1 cleavage and PAR formation; it reduced the P-AMPK level, restored P-p70S6K activation, and blocked autophagy induction. Moreover, cells that were treated with RAPA markedly enhanced AS-induced PARP1 cleavage and PAR and AMPK activation and eliminated the phosphorylation of p70S6K to induce autophagy.

Similar effects were also found in the NIT-1 cells with PS+DPQ, PS+BAF-A1, and PS+RAPA (*P* < 0.05, Figure 6(b)). The results show that PARP1 exhibits dual roles in changing the outcome of NIT-1 cells in response to AS. PARP1 activation is the cause of necrotic cell death through ATP depletion, and PARP1 activation can elicit a self-protective mechanism through the induction of autophagy via the AMPK-mTOR signaling pathway; however, necrotic cell death still eventually occurs. PARP1-AMPK activation is an important prosurvival mechanism in PS-treated cells through the suppression of mTOR and activation of autophagy.

### 3.7. Confocal Microscopic Studies on AS-Induced Mitochondrial Morphologic Changes in NIT-1 Cells

**Figure 7:**
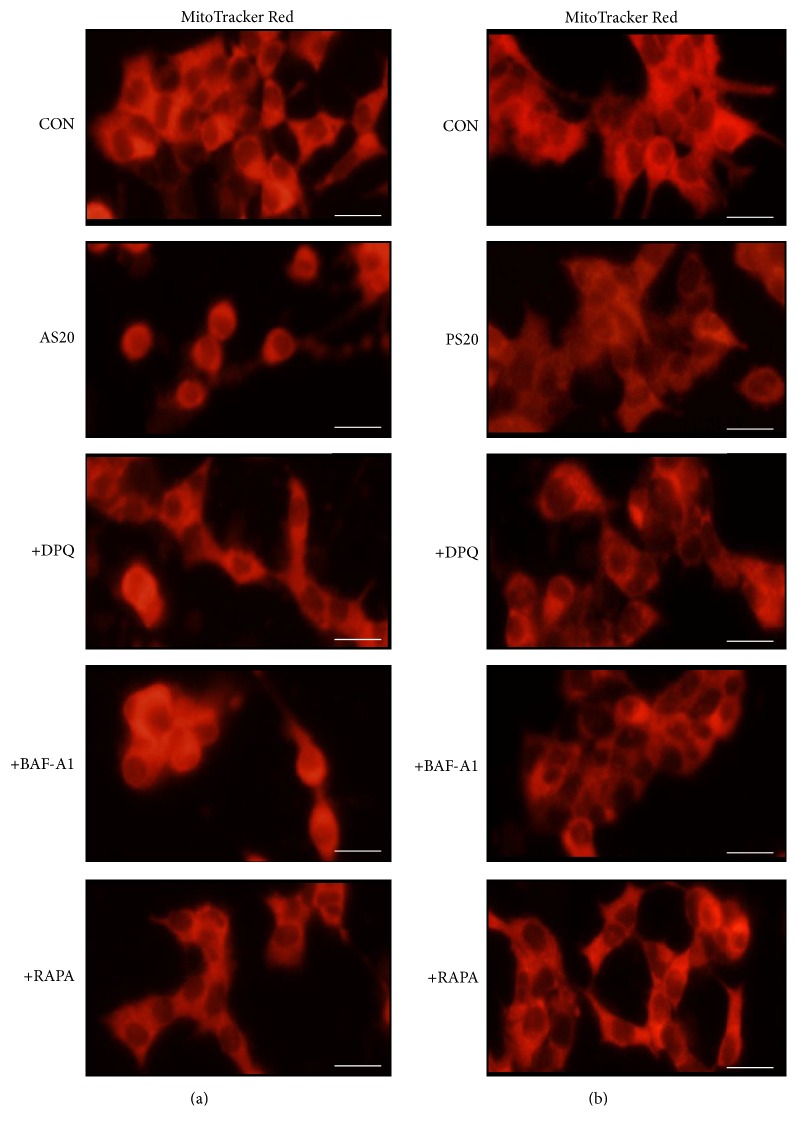
Confocal microscopic studies on AS-induced mitochondrial morphologic changes in NIT-1 cells. Cells were exposed to 20 *μ*M AS or 20 *μ*M PS with or without DPQ, BAF-A1, and RAPA pretreatment and stained with MitoTracker Red. After 48 h of AS treatment (a), the cells exhibited substantially less red fluorescent staining and mitochondria than the control (decreased 0.6-fold) and contained round, discrete mitochondria and diffused weak cytoplasmic fluorescence, which increased red fluorescence and improved mitochondrial morphology after DPQ treatment. PS-treated cells (b) exhibited mildly decreased red fluorescence and mitochondria compared with the control (decreased 0.1-fold) and showed improved diffused weak cytoplasmic fluorescence after DPQ treatment. Magnification: ×40. Scale bar: 20 *μ*M.

We examined the mitochondrial status of the AS- and PS-treated cells conjugated with MitoTracker Red; the accumulation of the cells is dependent on the membrane potential, and the cells are fusible into actively respiring cells. The AS- and PS-treated cells showed reduced red fluorescence intensity with fewer mitochondria; for the AS-treated cells, the reduction was approximately 0.5-fold relative to the control, and they contained round, discrete mitochondria and widely diffused weak cytoplasmic fluorescence (Figure 7, Figure S3B). Similar effects were also found in NIT-1 cells treated with AS+BAF-A1 and PS+BAF-A1. DPQ and RAPA pretreatment to some extent protected against AS- and PS-induced leakage of MitoTracker staining and substantially enhanced the mitochondrial morphology and staining intensity in NIT-1 cells. These results indicated that AS treatment reduced MitoTracker staining, thereby reducing mitochondrial activity; this explains the close association between PARP1 activation and mTOR suppression.

## 4. Discussion

The detailed mechanisms of the increased risk for developing diabetes during statin therapy remain unclear. We used the mouse NIT-1 insulinoma cell line as a pancreatic *β*-cell model, which is an effective tool for analyzing *β*-cell function and apoptosis [35, 36]. To examine whether statins mediate the outcome of NIT-1 cells, we used chemical inhibitor reagents to inhibit the expression of the target gene in NIT-1 cells. Our study results showed that atorvastatin treatment markedly reduced cell viability compared to untreated NIT-1 cells through increasing PARP1 activation and subsequently inducing necrosis and autophagy induction. This finding confirms previous findings that atorvastatin induced autophagic activity* in vivo* and* in vitro* [37–40]. In 2012, Lim et al. demonstrated that pravastatin can improve renal function in CsA-induced autophagic cell death through reducing LC3-II and P62 expression. Therefore, pravastatin may be associated with autophagy [41]. In this paper, we find that pravastatin treatment may increase PARP1 activation and immediately elicit a basal self-protective autophagy mechanism in conservative NIT-1 cells under pharmaceutical stress. In contrast, other studies showed that pravastatin did not result in induced autophagy activation in ovarian cancer cell, smooth muscle cell, and human rhabdomyosarcoma cell [42–44]. The reason for the differences observed is that the role of pravastatin in autophagy activation and function are cell type-specific. In this study, PARP1 exhibited dual roles in regulating the cell outcome in response to AS or PS treatment. Furthermore, this study revealed how statins induce necrosis and autophagy. The findings provide a reasonable basis for the future improvement of statin-based cardiovascular disease therapies.

### 4.1. Death of NIT-1 Cells Induced by Atorvastatin Is Not Apoptotic

Apoptosis has been considered to be the predominant mechanism induced by free fatty acids, cytokines, and glucose in NIT-1 cells [36, 45, 46]. In addition, several studies have shown that AS and PS could trigger different cells to mediate apoptosis* in vitro* and regulate cell growth* in vivo* [47–50]. We found that cell death was not inhibited by the pan-caspase inhibitor zVAD-fmk in NIT-1 cells treated with AS. The lack of apoptosis was further indicated by the absence of the apoptotic phase (Annexin V^+^/PI^−^). Given that AS killed 50%–60% of NIT-1 cells, these observations suggest that the effect of AS-induced NIT-1 cell death may not depend on apoptosis. According to the aforementioned studies, more than 50% of bladder cancer cells, T24, died 2-3 days after AS treatment (30 *μ*M) via activated caspase 3 and cleaved PARP [51]. In another study, increased Annexin V-positive cells and accumulated sub-G1 cell fractions were observed on day 3 after AS treatment with an increased dose (100 *μ*M), whereas AS treatment at 10 *μ*M showed a protective trend against apoptosis [52]. Thus, mechanisms other than apoptosis might exist in AS-mediated cell death, such as necrosis.

### 4.2. Autophagy Controls PARP1 Activation and Inhibits Necrosis, Leading to Enhanced Cell Survival

In recent years, the crosstalk among autophagy, apoptosis, and necrosis has been intensively studied. Most studies showed that autophagy is cytoprotective in cells under stress and inhibits apoptosis and necrosis [21, 53–56]. Moreover, necrosis is found to be typically accompanied by autophagy, but how autophagy counteracts necrosis and why autophagy cannot protect against AS-induced cell death remain unclear. We found that AS induces autophagy and necrotic cell death mediated by the activation of PARP1 and ROS. PARP1 may represent a master switch between cell death and cell survival.

A major finding in the present study is the crucial role of AMPK in AS- and PS-induced autophagy and cell death, as well as the downstream of PARP1 activation. AMPK is a highly conserved cellular energy sensor, and it is activated under stress conditions such as heat shock, hypoxia, ischemia, and glucose starvation [39, 57, 58]. Some evidence suggests that autophagy induction is regulated through AMPK-dependent phosphorylation, which leads to the inactivation of mTOR for homeostatic mitochondria and promotes cell survival [40, 59, 60]. Consistent with these findings, our results revealed that the activation of AMPK leads to the suppression of mTOR and induction of autophagy in NIT-1 cells exposed to AS and PS. Moreover, this study clearly suggested that autophagy is a cell survival mechanism in AS- and PS-mediated cell death, based on the suppression of autophagy caused by BAF-A1. By contrast, the activation of autophagy caused by rapamycin protected the AS- and PS-mediated cell death. Therefore, our findings suggest that targeting autophagy or PARP1-AMPK-mTOR pathways should be considered in the development of effective statin therapies for diabetes.

**Figure 8:**
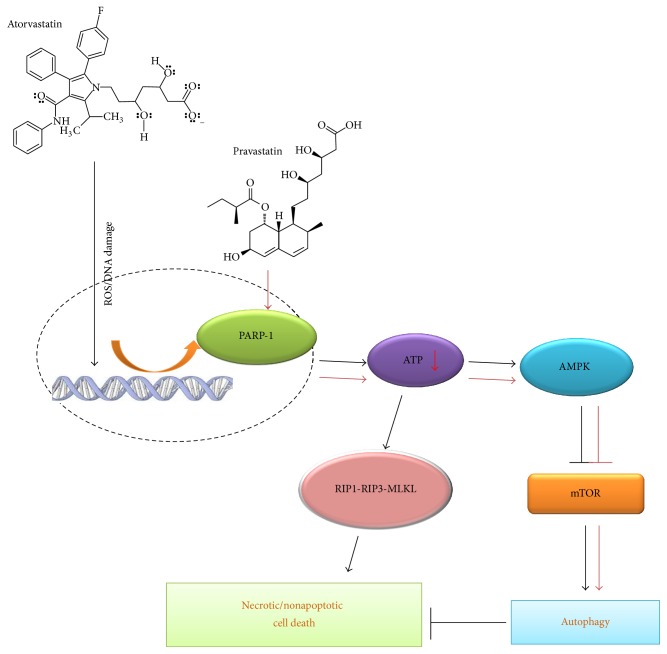
Illustration of the mechanisms of AS- and PS-induced autophagy and cell death. AS- and PS-induced autophagy and cell death are mediated by PARP1 activation, ATP depletion, AMPK activation, and mTOR and RIP1-RIP3-MLKL suppression.

In conclusion, based on a literature review, this study was the first to demonstrate a novel function of PARP1 in the regulation of AS- and PS-induced autophagy via the PARP1-AMPK-mTOR signaling pathway and that such autophagy serves as a cell survival mechanism against AS-mediated necrosis, although it is insufficient to prevent necrosis-induced cell death (Figure 8). These findings contribute to the understanding of the complex relationship among statin-related diabetes, autophagy, and cell death.

## Supplementary Material

Figure S1. Fluorescence staining of parthanatos marker AIF in NIT-1 cells treated with or without DPQ. Cell viability and LDH activity analysis in NIT-1 cells treated with or without DPQ, BAF-A1 and RAPA.
Figure S2. Fluorescence staining of autophagy marker LC3-II in NIT-1 cells treated with or without DPQ, BAF-A1 and RAPA. ATP concentration measurement in NIT-1 cells treated with or without DPQ, BAF-A1 and RAPA.
Figure S3. Western blot analysis of PARP1-AMPK-mTOR pathway in NIT-1 cells treated with or without DPQ, BAF-A1 and RAPA. Fluorescence staining of mitochondrial marker MitoTrackRed in NIT-1 cells treated with or without DPQ, BAF-A1 and RAPA.

## References

[1] Baigent C., Blackwell L., Emberson J. (2010). Efficacy and safety of more intensive lowering of LDL cholesterol: a meta-analysis of data from 170,000 participants in 26 randomised trials. *The Lancet*.

[2] Smith S. C., Allen J., Blair S. N. (2006). AHA/ACC guidelines for secondary prevention for patients with coronary and other atherosclerotic vascular disease: 2006 update endorsed by the National Heart, Lung, and Blood Institute. *Journal of the American College of Cardiology*.

[3] Kalaitzidis R. G., Elisaf M. S. (2011). The role of statins in chronic kidney disease. *American Journal of Nephrology*.

[4] Riganti C., Doublier S., Costamagna C. (2008). Activation of nuclear factor-*κ*B pathway by simvastatin and RhoA silencing increases doxorubicin cytotoxicity in human colon cancer HT29 cells. *Molecular Pharmacology*.

[5] Pelaia G., Gallelli L., Renda T. (2012). Effects of statins and farnesyl transferase inhibitors on ERK phosphorylation, apoptosis and cell viability in non-small lung cancer cells. *Cell Proliferation*.

[6] Mohammed A., Qian L., Janakiram N. B., Lightfoot S., Steele V. E., Rao C. V. (2012). Atorvastatin delays progression of pancreatic lesions to carcinoma by regulating PI3/AKT signaling in p48 Cre/+ LSL-Kras G12D/+ mice. *International Journal of Cancer*.

[7] Singh S., Singh A. G., Singh P. P., Murad M. H., Iyer P. G. (2013). Statins are associated with reduced risk of esophageal cancer, particularly in patients with Barrett's esophagus: a systematic review and meta-analysis. *Clinical Gastroenterology and Hepatology*.

[8] Collins R., Armitage J., Parish S. (2003). MRC/BHF Heart Protection Study of cholesterol-lowering with simvastatin in 5963 people with diabetes: a randomised placebo-controlled trial. *The Lancet*.

[9] Sever P. S., Dahlöf B., Poulter N. R. (2004). Prevention of coronary and stroke events with atorvastatin in hypertensive patients who have average or lower-than average cholesterol concentrations, in the Anglo-Scandinavian Cardiac Outcomes Trial–Lipid Lowering Arm (ASCOT-LLA): a multicentre randomised controlled trial. *Drugs*.

[10] Ridker P. M., Danielson E., Fonseca F. A. H. (2008). Rosuvastatin to prevent vascular events in men and women with elevated C-reactive protein. *The New England Journal of Medicine*.

[11] Sattar N., Preiss D., Murray H. M. (2010). Statins and risk of incident diabetes: a collaborative meta-analysis of randomised statin trials. *The Lancet*.

[12] Brault M., Ray J., Gomez Y.-H., Mantzoros C. S., Daskalopoulou S. S. (2014). Statin treatment and new-onset diabetes: a review of proposed mechanisms. *Metabolism*.

[13] Ruscica M., MacChi C., Morlotti B., Sirtori C. R., Magni P. (2014). Statin therapy and related risk of new-onset type 2 diabetes mellitus. *European Journal of Internal Medicine*.

[14] Baker W. L., Talati R., White C. M., Coleman C. I. (2010). Differing effect of statins on insulin sensitivity in non-diabetics: a systematic review and meta-analysis. *Diabetes Research and Clinical Practice*.

[15] Mita T., Watada H., Nakayama S. (2007). Preferable effect of pravastatin compared to atorvastatin on beta cell function in Japanese early-state type 2 diabetes with hypercholesterolemia. *Endocrine Journal*.

[16] Yamamoto H., Uchigata Y., Okamoto H. (1981). Streptozotocin and alloxan induce DNA strand breaks and poly(ADP-ribose) synthetase in pancreatic islets. *Nature*.

[17] Okamoto H., Takasawa S. (2003). Recent advances in physiological and pathological significance of NAD+ metabolites: roles of poly(ADP-ribose) and cyclic ADP-ribose in insulin secretion and diabetogenesis. *Nutrition Research Reviews*.

[18] Sims J. L., Berger S. J., Berger N. A. (1983). Poly(ADP-ribose) polymerase inhibitors preserve oxidized nicotinamide adenine dinucleotide and adenosine 5′-triphosphate pools in DNA-damaged cells: mechanism of stimulation of unscheduled DNA synthesis. *Biochemistry*.

[19] Ha H. C., Snyder S. H. (1999). Poly(ADP-ribose) polymerase is a mediator of necrotic cell death by ATP depletion. *Proceedings of the National Academy of Sciences of the United States of America*.

[20] Huang Q., Wu Y.-T., Tan H.-L., Ong C.-N., Shen H.-M. (2009). A novel function of poly(ADP-ribose) polymerase-1 in modulation of autophagy and necrosis under oxidative stress. *Cell Death and Differentiation*.

[21] Shen H.-M., Codogno P. (2012). Autophagy is a survival force via suppression of necrotic cell death. *Experimental Cell Research*.

[22] Zhou J., Ng S., Huang Q. (2013). AMPK mediates a pro-survival autophagy downstream of PARP-1 activation in response to DNA alkylating agents. *FEBS Letters*.

[23] Sarkar S., Perlstein E. O., Imarisio S. (2007). Small molecules enhance autophagy and reduce toxicity in Huntington's disease models. *Nature Chemical Biology*.

[24] Pickford F., Masliah E., Britschgi M. (2008). The autophagy-related protein beclin 1 shows reduced expression in early Alzheimer disease and regulates amyloid *β* accumulation in mice. *The Journal of Clinical Investigation*.

[25] Bergamini E., Cavallini G., Donati A., Gori Z. (2007). The role of autophagy in aging: its essential part in the anti-aging mechanism of caloric restriction. *Annals of the New York Academy of Sciences*.

[26] Liang X. H., Jackson S., Seaman M. (1999). Induction of autophagy and inhibition of tumorigenesis by beclin 1. *Nature*.

[27] Mathew R., Kongara S., Beaudoin B. (2007). Autophagy suppresses tumor progression by limiting chromosomal instability. *Genes & Development*.

[28] Baehrecke E. H. (2005). Autophagy: dual roles in life and death?. *Nature Reviews. Molecular Cell Biology*.

[29] Xu Y., Sung O. K., Li Y., Han J. (2006). Autophagy contributes to caspase-independent macrophage cell death. *Journal of Biological Chemistry*.

[30] Wu Y.-T., Tan H.-L., Huang Q. (2008). Autophagy plays a protective role during zVAD-induced necrotic cell death. *Autophagy*.

[31] Kroemer G., Levine B. (2008). Autophagic cell death: the story of a misnomer. *Nature Reviews Molecular Cell Biology*.

[32] Fatokun A. A., Dawson V. L., Dawson T. M. (2014). Parthanatos: mitochondrial-linked mechanisms and therapeutic opportunities. *British Journal of Pharmacology*.

[33] Wang Y., Dawson V. L., Dawson T. M. (2009). Poly(ADP-ribose) signals to mitochondrial AIF: a key event in parthanatos. *Experimental Neurology*.

[34] Jung C. H., Ro S.-H., Cao J., Otto N. M., Kim Do-Hyung D.-H. (2010). MTOR regulation of autophagy. *FEBS Letters*.

[35] Hamaguchi K., Gaskins H. R., Leiter E. H. (1991). NIT-1, a pancreatic *β*-cell line established from a transgenic NOD/Lt mouse. *Diabetes*.

[36] Augstein P., Heinke P., Salzsieder E. (2008). Dominance of cytokine- over FasL-induced impairment of the mitochondrial transmembrane potential (Δ*ψ*
_m_) in the pancreatic *β*-cell line NIT-1. *Diabetes and Vascular Disease Research*.

[37] Wang W., Wang H., Geng Q.-X. (2015). Augmentation of autophagy by atorvastatin via Akt/mTOR pathway in spontaneously hypertensive rats. *Hypertension Research*.

[38] Parikh A., Childress C., Deitrick K., Lin Q., Rukstalis D., Yang W. (2010). Statin-induced autophagy by inhibition of geranylgeranyl biosynthesis in prostate cancer PC3 cells. *Prostate*.

[39] Yang P.-M., Liu Y.-L., Lin Y.-C., Shun C.-T., Wu M.-S., Chen C.-C. (2010). Inhibition of autophagy enhances anticancer effects of atorvastatin in digestive malignancies. *Cancer Research*.

[40] Zhang Q., Yang Y.-J., Wang H. (2012). Autophagy activation: a novel mechanism of atorvastatin to protect mesenchymal stem cells from hypoxia and serum deprivation via AMP-activated protein kinase/mammalian target of rapamycin pathway. *Stem Cells and Development*.

[41] Lim S. W., Hyoung B. J., Piao S. G., Doh K. C., Chung B. H., Yang C. W. (2012). Chronic cyclosporine nephropathy is characterized by excessive autophagosome formation and decreased autophagic clearance. *Transplantation*.

[42] Robinson E., Nandi M., Wilkinson L. L., Arrowsmith D. M., Curtis A. D. M., Richardson A. (2013). Preclinical evaluation of statins as a treatment for ovarian cancer. *Gynecologic Oncology*.

[43] Martinet W., Schrijvers D. M., Timmermans J.-P., Bult H. (2008). Interactions between cell death induced by statins and 7-ketocholesterol in rabbit aorta smooth muscle cells. *British Journal of Pharmacology*.

[44] Araki M., Motojima K. (2008). Hydrophobic statins induce autophagy in cultured human rhabdomyosarcoma cells. *Biochemical and Biophysical Research Communications*.

[45] Zhang Y., Xu M., Zhang S. (2007). The role of G protein-coupled receptor 40 in lipoapoptosis in mouse *β*-cell line NIT-1. *Journal of Molecular Endocrinology*.

[46] Yuan H., Lu Y., Huang X. (2010). Suppression of NADPH oxidase 2 substantially restores glucose-induced dysfunction of pancreatic NIT-1 cells. *FEBS Journal*.

[47] Cheng C.-F., Juan S.-H., Chen J.-J. (2008). Pravastatin attenuates carboplatin-induced cardiotoxicity via inhibition of oxidative stress associated apoptosis. *Apoptosis*.

[48] Aprigliano I., Dudas J., Ramadori G., Saile B. (2008). Atorvastatin induces apoptosis by a caspase-9-dependent pathway: an in vitro study on activated rat hepatic stellate cells. *Liver International*.

[49] Song X. J., Yang C. Y., Liu B. (2011). Atorvastatin inhibits myocardial cell apoptosis in a rat model with post-myocardial infarction heart failure by downregulating ER stress response. *International Journal of Medical Sciences*.

[50] Qi X.-F., Zheng L., Lee K.-J. (2013). HMG-CoA reductase inhibitors induce apoptosis of lymphoma cells by promoting ROS generation and regulating Akt, Erk and p38 signals via suppression of mevalonate pathway. *Cell Death and Disease*.

[51] Kang M., Jeong C. W., Ku J. H., Kwak C., Kim H. H. (2014). Inhibition of autophagy potentiates atorvastatin-induced apoptotic cell death in human bladder cancer cells in vitro. *International Journal of Molecular Sciences*.

[52] Klein S., Klösel J., Schierwagen R. (2012). Atorvastatin inhibits proliferation and apoptosis, but induces senescence in hepatic myofibroblasts and thereby attenuates hepatic fibrosis in rats. *Laboratory Investigation*.

[53] González-Polo R.-A., Boya P., Pauleau A.-L. (2005). The apoptosis/autophagy paradox: autophagic vacuolization before apoptotic death. *Journal of Cell Science*.

[54] Scarlatti F., Granata R., Meijer A. J., Codogno P. (2009). Does autophagy have a license to kill mammalian cells?. *Cell Death and Differentiation*.

[55] Esposti D. D., Domart M.-C., Sebagh M. (2010). Autophagy is induced by ischemic preconditioning in human livers formerly treated by chemotherapy to limit necrosis. *Autophagy*.

[56] Amelio I., Melino G., Knight R. A. (2011). Cell death pathology: cross-talk with autophagy and its clinical implications. *Biochemical and Biophysical Research Communications*.

[57] Hardie D. G. (2004). The AMP-activated protein kinase pathway—new players upstream and downstream. *Journal of Cell Science*.

[58] Hardie D. G. (2007). AMP-activated/SNF1 protein kinases: conserved guardians of cellular energy. *Nature Reviews Molecular Cell Biology*.

[59] Liang J., Shao S. H., Xu Z.-X. (2007). The energy sensing LKB1-AMPK pathway regulates p27kip1 phosphorylation mediating the decision to enter autophagy or apoptosis. *Nature Cell Biology*.

[60] Alers S., Löffler A. S., Wesselborg S., Stork B. (2012). Role of AMPK-mTOR-Ulk1/2 in the regulation of autophagy: cross talk, shortcuts, and feedbacks. *Molecular and Cellular Biology*.

