# Supplementary figures and images for: The Different Effects of Atorvastatin and Pravastatin on Cell Death and PARP Activity in Pancreatic NIT-1 Cells
Source: J Diabetes Res. 2016 Nov 27;2016:1828071. doi: 10.1155/2016/1828071 (PMC5149701; doi:10.1155/2016/1828071)

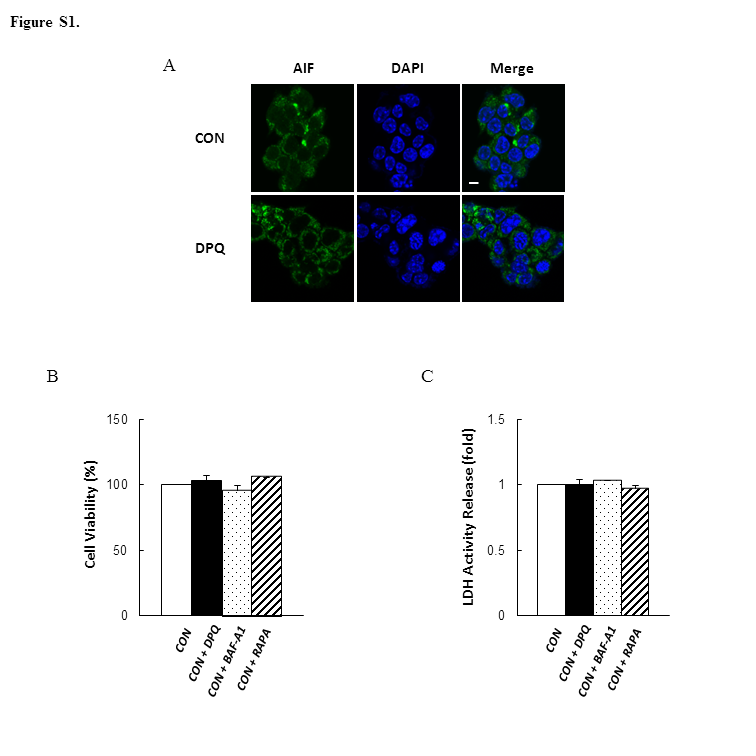


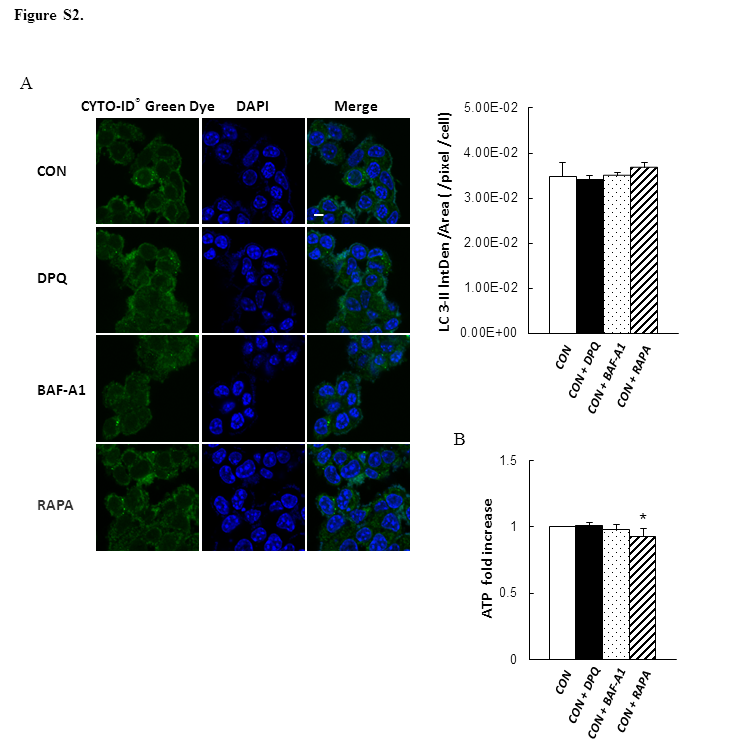


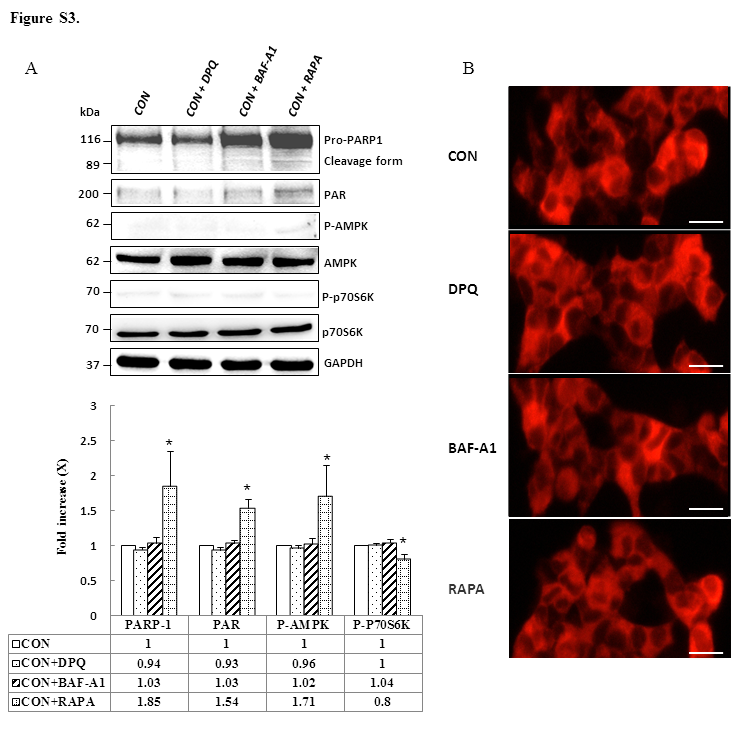

Supplement: Supplementary file 1 — Figure S1. Fluorescence staining of parthanatos marker AIF in NIT-1 cells treated with or without DPQ. Cell viability and LDH activity analysis in NIT-1 cells treated with or without DPQ, BAF-A1 and RAPA. Figure S2. Fluorescence staining of autophagy marker LC3-II in NIT-1 cells treated with or without DPQ, BAF-A1 and RAPA. ATP concentration measurement in NIT-1 cells treated with or without DPQ, BAF-A1 and RAPA. Figure S3. Western blot analysis of PARP1-AMPK-mTOR pathway in NIT-1 cells treated with or without DPQ, BAF-A1 and RAPA. Fluorescence staining of mitochondrial marker MitoTrackRed in NIT-1 cells treated with or without DPQ, BAF-A1 and RAPA. [file 1828071.f1.docx]
